# Comparative analysis of *Vibrio cholerae* isolates from Ghana reveals variations in genome architecture and adaptation of outbreak and environmental strains

**DOI:** 10.3389/fmicb.2022.998182

**Published:** 2022-10-13

**Authors:** Nana Eghele Adade, Yaw Aniweh, Lydia Mosi, Miguel A. Valvano, Samuel Duodu, Stephen Dela Ahator

**Affiliations:** ^1^West African Centre for Cell Biology of Infectious Pathogens, College of Basic and Applied Sciences, University of Ghana, Accra, Ghana; ^2^Department of Biochemistry, Cell, and Molecular Biology, College of Basic and Applied Sciences, University of Ghana, Accra, Ghana; ^3^Wellcome-Wolfson Institute for Experimental Medicine, Queen’s University Belfast, Belfast, United Kingdom; ^4^Department of Microbiology, Korle-Bu Teaching Hospital, Accra, Ghana; ^5^Centre for New Antibacterial Strategies (CANS) and Research Group for Host-Microbe Interactions, Department of Medical Biology, Faculty of Health Sciences, UiT- The Arctic University of Norway, Tromsø, Norway

**Keywords:** *Vibrio cholerae*, genome plasticity, genetic recombination, CRISPR-Cas system, phage and plasmid defense, pseudogenes

## Abstract

Recurrent epidemics of cholera denote robust adaptive mechanisms of *Vibrio cholerae* for ecological shifting and persistence despite variable stress conditions. Tracking the evolution of pathobiological traits requires comparative genomic studies of isolates from endemic areas. Here, we investigated the genetic differentiation among *V. cholerae* clinical and environmental isolates by highlighting the genomic divergence associated with gene decay, genome plasticity, and the acquisition of virulence and adaptive traits. The clinical isolates showed high phylogenetic relatedness due to a higher frequency of shared orthologs and fewer gene variants in contrast to the evolutionarily divergent environmental strains. Divergence of the environmental isolates is linked to extensive genomic rearrangements in regions containing mobile genetic elements resulting in numerous breakpoints, relocations, and insertions coupled with the loss of virulence determinants *acf*, *zot*, *tcp*, and *ctx* in the genomic islands. Also, four isolates possessed the CRISPR-Cas systems with spacers specific for *Vibrio* phages and plasmids. Genome synteny and homology analysis of the CRISPR-Cas systems suggest horizontal acquisition. The marked differences in the distribution of other phage and plasmid defense systems such as Zorya, DdmABC, DdmDE, and type-I Restriction Modification systems among the isolates indicated a higher propensity for plasmid or phage disseminated traits in the environmental isolates. Our results reveal that *V. cholerae* strains undergo extensive genomic rearrangements coupled with gene acquisition, reflecting their adaptation during ecological shifts and pathogenicity.

## Introduction

*Vibrio cholerae*, which causes the life-threatening diarrheal disease cholera and is responsible for an ongoing pandemic since 1961, can reside in multiple hosts and transition from non-pathogenic to pathogenic states (Domman et al., 2017). *V. cholerae* can be often isolated from aquatic environments and as a facultative human pathogen (Mohamad et al., 2019; Islam et al., 2020). They are Gram-negative curved rod bacteria comprising over 200 serogroups, with only two serogroups (O1 and O139) implicated in epidemic and pandemic cholera (Yamai et al., 1997; Domman et al., 2017). The O1 serogroup is further classified into El-Tor and Classical biotypes (Kaper et al., 1995).

*Vibrio cholerae* isolates harbor two circular chromosomes, with few exceptions with a single fused chromosome (Okada et al., 2005; Johnson et al., 2015; Bruhn et al., 2018; Sozhamannan and Waldminghaus, 2020). The genomic architecture of *V. cholerae* is influenced by its lifestyle, which explains the evolution of adaptive traits and acquisition or loss of virulence determinants enabling switching from a non-pathogenic state in the environment to a pathogenic state during infection in humans. Continuous adaptive evolution of the *V. cholerae* strains is attributed to genetic recombination events and genomic rearrangements, as well as loss or acquisition of genomic segments *via* horizontal gene transfer (HGT) (Grim et al., 2010; Keymer and Boehm, 2011; Devault et al., 2014). Variations in the genomes of *V. cholerae* strains are influenced by horizontally acquired mobile genetic elements (MGEs) that have distinct composition compared to the whole genome. Genomic islands (GIs), composed of MGEs, are distributed in the genomes of pathogenic and non-pathogenic *V. cholerae* strains and contribute to genomic variability and clonal diversity; they also encode functions that drive adaptation (Dobrindt et al., 2004; Pant et al., 2020). Most pathogenic *V. cholerae* isolates harbor multiple prophages (CTXΦ, RS1Φ, TLCΦ), GIs (VPI-1, VPI-2, VSP-1, VSP-2), and the integrative conjugative element (ICE) (Weill et al., 2019; Pant et al., 2020). Infections and subsequent illnesses caused by *V. cholerae* depend on pathogenicity factors such as cholerae toxin (Ctx), toxin coregulated proteins (Tcp), and accessory colonizing factors (Acf) that are encoded in mobile GIs.

Concerning genetic material acquisition, natural competence to obtain environmental DNA, which may be heavily degraded or originate from less fit bacteria, can be less effective to capture coding regions favorable for *V. cholerae* (Søren et al., 2013; Matthey et al., 2019). Hence, *V. cholerae* can gain fitness advantages by acquiring beneficial genetic traits *via* HGT or lateral gene transfer from phages or other bacteria *via* kin-selection and neighbor predation by the Type VI (T6SS)-mediated duels (Ho et al., 2014). Most of the genes encoding the T6SS core components are usually located in the GIs of both pathogenic and non-pathogenic *V. cholerae* strains. The coupling of competence and the T6SS is highly conserved in several non-cholera vibrios (Metzger et al., 2019) and is controlled by TfoX, HapR, Dns, and QstR regulatory proteins (Metzger et al., 2016; Jaskólska et al., 2018). Additionally, numerous pathogenic and non-pathogenic *V. cholerae* strains possess active Clustered Regularly Interspaced Short Palindromic Repeats (CRISPR) and CRISPR-associated (Cas) proteins, which interfere with the acquisition of non-beneficial genetic segments and defend against phage infections.

There is much to learn about *V. cholerae* survival in the environment during interepidemic periods and the adaptive mechanisms for surviving against biotic and abiotic stress in the environment. Investigating the variation in genome architecture between environmental and clinical isolates, which may enable the transition from the non-pathogenic state to the pathogenic state, can add significant understanding to potentially control recurring epidemics. Here, we report the isolation and genomic analysis of *V. cholerae* outbreak (clinical) and environmental isolates from Ghana. We conducted a systematic comparative genomic analysis on *V. cholerae* isolates and elucidated pathoadaptive mutations arising from genomic rearrangements in these isolates. Our analysis further revealed genetic clusters that drive adaptation of clinical and environmental strains during infection and survival in the ecological niche, respectively. We examined the presence of classical virulence determinants encoded by the *ctx*, *zot*, *tcp*, and *acf* gene clusters and the genomic context of the CRISPR-Cas systems in the isolates in terms of their acquisition as single modules or within GIs. Additionally, single nucleotide polymorphism (SNP) and ortholog-based phylogenetic analyses illustrated the relatedness, progressive evolution, and conservation of genomic content among the isolates. We further describe the genomic variability arising due to the presence of MGEs, recombination patterns, gene decay, and genome rearrangement between the *V. cholerae* isolates.

## Materials and methods

### Isolation and sources of *Vibrio cholerae* strains

The *V. cholerae* strains S1, S35, E4, E19, E30, and E32 were isolated from the Korle-Lagoon in the Greater Accra region of Ghana from 2016 to 2020. The clinical or outbreak strains; C23, C22, C14, C1, C6, and C17 were collected between 2010 and 2015 from the Public Health and Reference laboratory (NHPRL) in the Greater Accra region of Ghana.

### Whole-genome sequencing

Genomic DNA was extracted using the QIAamp DNA Mini Kit, as instructed by the manufacturer and quantified using NanoDrop. Genome sequencing was done by MicrobesNG (Birmingham); the genomic DNA library was prepared using the Nextera XT library prep kit (Illumina) with the following modifications: two nanograms of DNA instead of one were used as input and PCR elongation time was increased to 1 min from 30 s. DNA quantification and library preparation were carried out on a Hamilton Microlab STAR automated liquid handling system. Pooled libraries were quantified using the Kapa Biosystems Library Quantification Kit for Illumina on a Roche light cycler 96 qPCR machine. Libraries were sequenced on the Illumina HiSeq using a 250 bp paired end protocol. Reads were adapter trimmed using Trimmomatic 0.30 (Bolger et al., 2014) with a sliding window quality cutoff of Q15 and the assembly matrix calculated with QUAST (Gurevich et al., 2013). *De novo* assembly was performed on samples using SPAdes version 3.7 (Nurk et al., 2013), and Contigs were annotated using Prokka 1.11 (Seemann, 2014) all *via* MicrobesNG (Birmingham). The RAST server was used for Gene-finding, annotation, and functional categorization (Seemann, 2014).

### Comparative genomics

Orthologs shared among the *V. cholerae* strains were identified using Orthofinder (Emms and Kelly, 2019). Genetic variants such as SNPs, MNPs, indels, and insertions were identified using SNIPPY (Seemann, 2015). Whole genome alignment for the identification of inversions, translocations, and breakpoints in the genomes was performed using MUMmer4 (Seemann, 2015). Visualization of the whole genome alignment and segments of the genomes was performed using the CGview server (Stothard et al., 2019), and Easyfig (Sullivan et al., 2011). The SIGI-HMM and IslandPath-DIMOB packages in IslandViewer 4 were used for the identification of genomic island (GI) (Bertelli et al., 2017). Mass screening of virulence and antimicrobial genes was performed using ABRicate (Seemann, 2016). Identification of recombination patterns in the genomes was conducted using Gubbins (Croucher et al., 2015) and visualized with Phandango (Croucher et al., 2015). The Integrative and Conjugative Elements (ICE) in the *V. cholerae* isolates were identified using ICEfinder (Liu et al., 2019).

### Identification of pseudogenes

Pseudogenes were predicted using a combination of Pseudofinder (Syberg-Olsen et al., 2021) and the MetaGeneAnnotator, and Prodigal packages in DFAST prokaryotic genome annotation pipeline (Tanizawa et al., 2018). Classification of pseudogenes was based on the presence of significant frameshift, indels, loss of stop or start codons, fragmentation, truncated or internal stop codons, partial gene deletions, and gene remnants found within intergenic regions.

### Identification of protospacer targets

The identification of protospacer targets of the type I-F, type I-E, and the mini type I-F CRISPR-Cas systems was performed using the CRISPRTarget program with a cut-off score of 25 [default 20, (26/32 basepairs)]. The Genbank-phage, IMGVR, RefSeq-Plasmid, PHAST, HuVirDB, and RefSeq-Viral databases were used for the protospacer search under the CRISPRTarget program (Biswas et al., 2013).

### Data availability

Sequence data for all strains used in this study can be found in NCBI with the Accession numbers: SAMN26804330, C23; SAMN26804331, C22; SAMN26804328, C14; SAMN26804326, C1; SAMN26804327, C6; SAMN26804329, C17; SAMN26804320, S1; SAMN26804321, S35; SAMN26804322, E4; SAMN26804323, E19; SAMN26804324, E30, and SAMN26804325, E30.

## Results

### General genomic features and divergence of *Vibrio cholerae* isolates

The clinical outbreak strains (C1, C6, C14, C17, C22, and C23) were collected between 2010 and 2015 from the Public Health and Reference Laboratory (NHPRL) in Accra, Ghana. The environmental isolates were sampled from the Korle-Lagoon in Accra, Ghana from 2016 to 2020. Except for one Classical biotype C6, all outbreak strains were El-Tor biotypes (Table 1). All isolates contained two circular chromosomes with a combined size ranging from 3.92 to 4.09 Mb and were composed of 3,443–3,627 coding sequences (Supplementary Figure 1 and Table 2). The genomes of the clinical and environmental isolates differed in the number of genomic islands (GIs) and the elements encoded in those regions (Supplementary Figure 1). Further analysis of the % GC content of the isolates revealed a range from 47.5 to 47.7 and almost similar average coding ratios among all the isolates (Table 1), indicating a high level of genome content conservation among them despite the differences in the source and period of isolation.

**TABLE 1:** Description of the *Vibrio cholerae* isolates obtained from stool and water samples.

Accession No	Strain ID	Year	Source	Isolate	Serogroup	Biotype	Tax ID	Source
SAMN26804330	C23	2014	Outbreak	*V. cholerae*	O1	El-Tor Ogawa	666	Stool
SAMN26804331	C22	2011	Outbreak	*V. cholerae*	O1	El-Tor Ogawa	666	Stool
SAMN26804328	C14	2015	Outbreak	*V. cholerae*	O1	El-Tor Ogawa	666	Stool
SAMN26804326	C1	2010	Outbreak	*V. cholerae*	O1	El-Tor Ogawa	666	Stool
SAMN26804327	C6	2010	Outbreak	*V. cholerae*	O1	Classical Ogawa	666	Stool
SAMN26804329	C17	2014	Outbreak	*V. cholerae*	O1	El-Tor Ogawa	666	Stool
SAMN26804320	S1	2019	Environmental	*V. cholerae*	non O1/O139	–	666	Water
SAMN26804321	*S35*	2020	Environmental	*V. cholerae*	non O1/O139	–	666	Water
SAMN26804322	E4	2016	Environmental	*V. cholerae*	non O1/O139	–	666	Water
SAMN26804323	E19.	2016	Environmental	*V. cholerae*	non O1/O139	–	666	Water
SAMN26804324	E30	2016	Environmental	*V. cholerae*	non O1/O139	–	666	Water
SAMN26804325	E32	2016	Environmental	*V. cholerae*	non O1/O139	–	666	Water

**TABLE 2:** General features of the *Vibrio cholerae* genomes.

General features of *Vibrio cholerae* strain genomes												
Vibrio isolates	C1	C6	C14	C17	C22	C23	E4	E19	E30	E32	S1	S35
Genome size (bp)	4090818	4039663	4041643	4089897	4088779	4091606	4053514	3968586	3968081	3968823	3979288	3920047
GC content (%)	47.5	47.5	47.5	47.5	47.5	47.5	47.5	47.7	47.6	47.6	47.6	47.6
Ribosomal RNA	9	12	10	9	10	10	14	11	11	11	8	13
Transfer RNAs	90	91	89	87	86	87	91	102	93	90	93	90
Completeness (%)	99.69	99.42	99.69	99.69	99.69	99.69	99.42	99.7	99.7	99.71	99.42	99.28
Predicted genes	3720	3731	3655	3712	3716	3715	3699	3583	3578	3582	3586	3547
Coding sequences (CDS)	3620	3627	3555	3615	3619	3617	3593	3468	3473	3481	3485	3443
Coding sequences present in all genomes	2874(79.4%)	2874(79.2%)	2874(80.8%)	2874(79.5%)	2874(79.4%)	2874(79.5%)	2874(80%)	2874(82.9%)	2874(82.8%)	2874(82.6%)	2874(82.5%)	2874(83.5%)
Coding ratio (%)	86.8	86.9	86.8	86.7	86.9	86.8	86.5	86.4	86.7	86.7	86.3	86.9
Pseudogenes	184	214	176	179	185	183	211	166	185	191	177	173
Complex variants (snp and mnp)	1	2385	3	1	1	1	5559	6839	6857	6958	5677	5586
Deletion regions (against N16961 genome)	21	110	26	21	21	22	166	198	204	208	195	164
Insertions regions (against N16961 genome)	6	81	6	6	6	6	170	192	186	189	162	154
Multiple nucleotide polymorphism (MNPs) (against N16961 genome)	0	173	0	0	0	0	626	686	654	506	336	513
Single nucleotide differences (SNPs) (against N16961 genome)	125	14837	148	124	125	124	36532	39934	39998	40007	35500	36219
Total variants (against N16961 genome)	153	17586	183	152	153	153	43053	47849	47899	47868	41870	42636
Breakpoints	485	709	521	551	487	516	1535	1443	1376	1321	1442	1417
Relocations	24	45	31	41	36	35	110	79	77	101	90	89
Translocations	33	21	26	18	19	26	13	34	28	12	26	18
Inversions	12	22	19	14	11	18	19	11	16	13	20	8
Insertions	40	116	47	47	42	37	574	409	350	328	445	460
Number of genes in orthogroups	3620	3497	3552	3615	3619	3617	3456	3361	3473	3481	3362	3312
Shared orthogroups	2981	2981	2981	2981	2981	2981	2981	2981	2981	2981	2981	2981
Unique or unassigned genes to orthogroups	0	130	3	0	0	0	137	108	3	0	122	131
Percentage of genes in orthogroups (%)	100	96.4	99.9	100	100	100	96.2	96.9	100	100	96.5	96.2
Number of CRISPR loci (number of repeats in each locus)	0	1(36)	0	0	0	0	1(39)	2(50 and 10)	0	0	1(3)	0
Complex variants = combination of snp/mnp												

Most genes in the isolates were classified as hypothetic or unknown function based on overall functional categorization. Environmental isolates possessed less genes in the categories of membrane transport, carbohydrates, Phage/prophage/transposable elements/plasmids, virulence/disease/defense, and stress response when compared to the clinical isolates (Supplementary Figure 2A). Also, there were less genes implicated in sulfur metabolism in environmental isolates than in clinical isolates. Differences in genes for the locus known as “clustered regularly interspaced short palindromic repeats” (CRISPR) arrays and the orientation of the CRISPR associated (*cas*) genes were observed in one clinical isolate (C6) and three environmental isolates (E4, E19, and S1) (Table 2). Two CRISPR arrays were in the integron genomic region of E19; however, only one was associated with Cas proteins.

**FIGURE 1:**
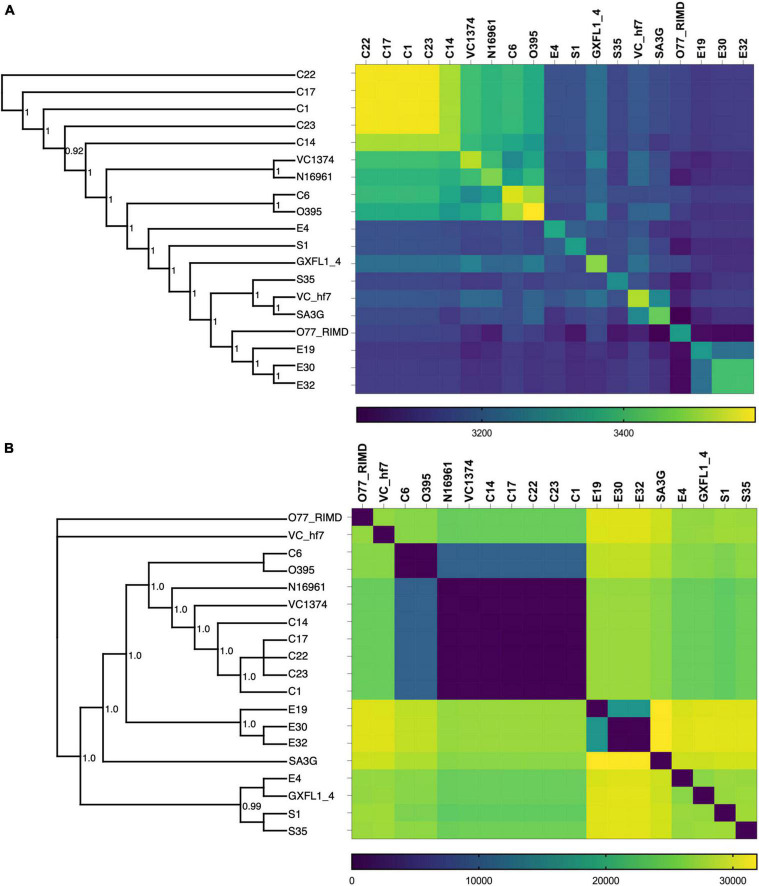
Ortholog and SNP-based phylogeny of the *V. cholerae* isolates. **(A)** Combined whole genome phylogenetic tree and Ortholog-based matrix showing the relatedness and divergence of the isolates based on the ortholog assignment of genes. **(B)** SNP-based phylogenetic tree and matrix showing the relationship between the clinical and environmental isolates. Included in the analysis are the *V. cholerae* strains N16961, VC1374, O395, GXFL-4, VC-hf7, SA3G, and O77 RIMD genomes obtained from the NCBI datasets.

To investigate the phylogenomic divergence and molecular evolution, we analyzed the shared orthologs and SNPs present in the genomes of the clinical and environmental isolates. Orthologs represent genes derived by vertical gene transfer from a common ancestor and often retain similar biological functions. From the rooted-species tree we observed high relatedness between the clinical isolates with greater numbers of shared orthologs (Figure 1A). The evolutionary tree derived from the ortholog-based phylogeny suggests that the environmental isolates were descendants of the clinical isolates (Figure 1A) and diverged relative to the period of isolation (Table 1). The clustering of ortholog-based phylogeny between the clinical and environmental isolates showed similarities with their SNP-based phylogeny (Figure 1B). More orthologs were shared among the clinical isolates than among the environmental isolates, indicating that the clinical isolates share and maintain substantial proportions of their overall genome content (Figure 1A). The percentage of genes that belonged to one or more orthogroups among the isolates was 98.5%. From the set of orthologous genes in the isolates, 2,981 (representing between 82 and 86% of genes) were shared (Table 2). Such high proportion of common orthologs shows that both the clinical and environmental strains share a substantial part of their overall genome contents. Aside from C1, C7, C22, C23, and E32, the rest of the isolates contained unique genes without assigned orthologs (Table 2). Most of the unassigned genes encoded hypothetical proteins while others encoded toxins, transposases, endonucleases, stress response, and virulence factors. Two putative xenologs were identified in three clinical isolates (C1, C17, and C22) and five environmental strains (E4, E30, E32, S1, and S35). They include genes encoding a LysR family transcriptional regulator (AAF94208.1) and a type II toxin-antitoxin system, RelB (WP_001258569.1). The LysR family regulator was commonly located in pathogenicity islands (McDonald et al., 2019). Likewise, the *relB* was found in the GI-2 (integron) (Supplementary Figure 1) of several *Vibrio* strains (Makino et al., 2003).

SNPs provide an ideal tool to explore genomes for adaptation to different niches and to investigate the local diversification of isolates. We observed more SNPs among the environmental isolates than clinical isolates (Figure 1B), which denotes the progressive evolution of environmental bacteria across the periods of isolation. This reflects exposure of environmental bacteria to diverse stress conditions (Makarova and Koonin, 2015), while the accumulation of mutations in the clinical isolates was constrained despite different isolation times. The SNP-based phylogenetic analysis indicated that the branch points and relatedness between the strains shared similarities that were estimated for orthologs (Figure 1A,B). However, the SNPs and ortholog distributions were inversely proportional, as isolates with high numbers of shared orthologs had fewer SNPs and vice versa (Figures 1A,B).

### Inactivation of *Vibrio cholerae* genes

The distribution of pseudogenes in a bacterial genome indicates the ecological shift experienced by bacteria and the selection against non-essential genes for survival in the new environments (Lerat and Ochman, 2005). Pseudogenization arises from indels or mutations that are triggered by impaired repair systems or SOS responses (Buysse et al., 2021), and result in the reduction of genes encoding non-redundant or overlapping functions (Makarova and Koonin, 2015). We investigated the presence of pseudogenes in the isolates with Pseudofinder, prodigal, and MetaGeneAnnotator (Syberg-Olsen et al., 2021). Most pseudogenes encoded hypothetical proteins or ORFs with unknown functions and insertion sequences (transposons and prophage or phage sequences) (Supplementary Table 1), and were more abundant in the GIs with characteristic high genome instability due to insertion and phage sequences. This implies that pseudogenes may have been acquired through HGT and could be jettisoned from the genome over time if they do not enhance the metabolic fitness or adaptation (Kuo and Ochman, 2010; Burke and Moran, 2011; Bennett and Moran, 2015; McCutcheon et al., 2019). Concerning potential mechanisms of pseudogenization, the clinical and environmental isolates had genes involved in mismatch repair (*mutHLSY*), recombination (*recABCDFNOQRX*), SOS response (*polB*, *dinB*, *lexA*), and excision repair (*uvrABCD*), which remained intact or not pseudogenized and were therefore deemed to be functional (Supplementary Table 2; Bennett and Moran, 2015; McCutcheon et al., 2019). In contrast, genes encoding the mutagenic polymerase UmuCD were absent or pseudogenized in some isolates (Supplementary Table 2). Although no clear link has been reported between the DNA polymerase V subunits *umuC* and *umuD* and the occurrence of pseudogenes, *umuCD* inactivation leads to increased recombination activity, unrepaired DNA lesions, and subsequent genetic variations (Makarova and Koonin, 2015).

### Analysis of virulence and pathogenic genes and pathways

Virulence traits are important for bacterial infection and survival in the host. The expression of factors such as toxin-coregulated pilus (TCP) and cholera toxins (CT) are essential for *V. cholerae* colonization in the small intestines and diarrhea, respectively (Thelin and Taylor, 1996). Further, the accessory colonization factor *acfABCD* gene cluster enables intestinal colonization and biogenesis of the toxin-associated pilus. The *tcp*, *acf*, and *ctx* gene clusters were absent in the environmental isolates, which also lacked the zonula occludens toxin gene *zot*, and a gene encoding the actin cross-linking toxin VgrG-3 (N16961_VCA02851) (Supplementary Table 3).

**FIGURE 2:**
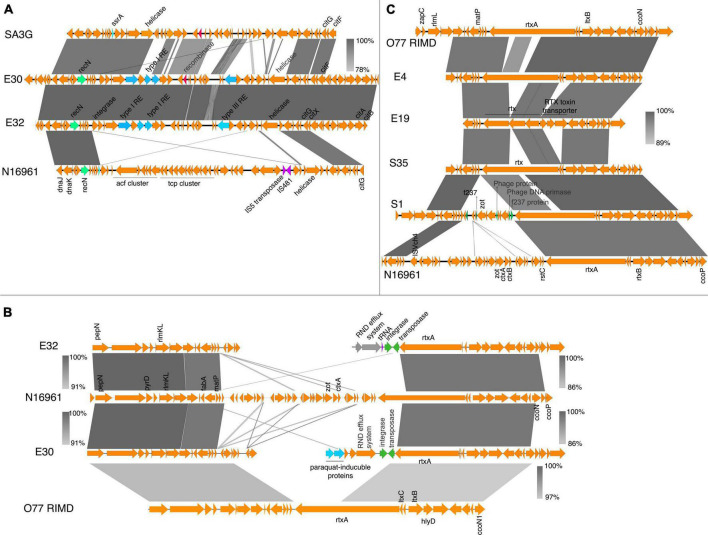
Virulence genes in *V. cholerae* isolates. **(A)** Schematic gene organization and alignments of the region containing the *acf* and *tcp* gene clusters in the N16961, SA3G, and the environmental isolates E30 and E32. The E30 and E32 share high similarities (between 93 and 100%) in gene organization around the location containing the virulence gene clusters in the reference strain. **(B)** Gene organization and alignment of the *zot, ctxAB* region in N16961, SA3G and the environmental isolates E30 and E32. **(C)** The gene order and alignment of the zot and *ctx* gene clusters in E4, E19, S35, S1, N16961, and O77 RIMD. The absence of the *zot* and *ctx* genes and disruption in synteny around the genomic regions is observed in the E30 and E32 isolates. The decay in the gene order in the *zot* and *ctx* region compared to the reference strain is shown by discontinuous regions of genomic fragments aligned above and below the N16961 gene cluster. The grayscale represents nucleotide homology.

**FIGURE 3:**
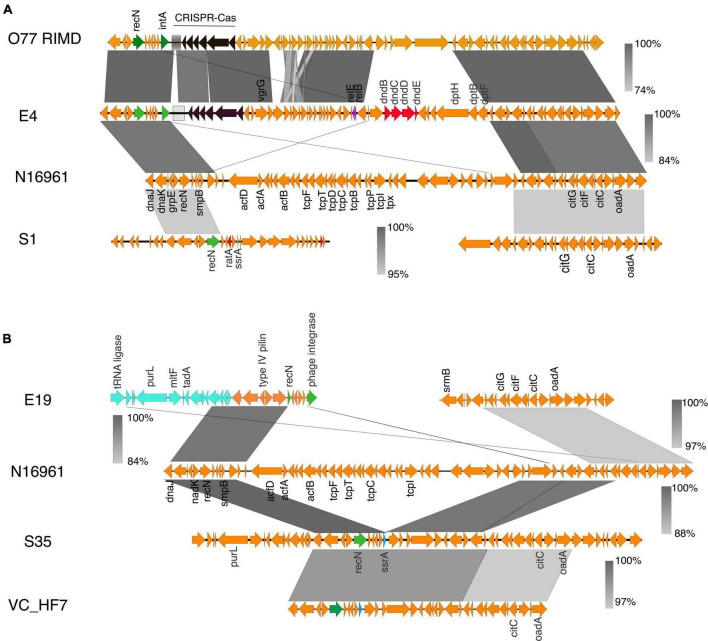
Virulence genes in *V. cholerae* isolates. **(A)** The gene organization and alignment of the *acf* and *tcp* region in the N16961, O77 RIMD and the environmental isolates S1 and E4. **(B)** Genetic organization of the *tcp* and *acf* gene cluster region in N16961, VC-hf7 and the isolates E19 and S35. The grayscale bars show the nucleotide homology in the aligned regions. Insertion elements, DNA metabolism genes, the phage sequences are in green.

The syntenic organization of the region containing *tcp*, *acf*, *zot*, and *ctx* revealed significant genomic rearrangements in the environmental isolates (Figures 2, 3). In contrast, synteny was conserved in the clinical isolates when compared with N16961 (Supplementary Figure 3), except for the Classical Ogawa isolate C6, which showed a decay in synteny and homology, particularly in the region containing the *zot* and *ctx* genes with the N16961 and the classical strain *V. cholerae* O395 (Supplementary Figure 4A). The *acf*, *ctx*, *tcp*, and *zot* clusters are in the genomic islands (Supplementary Figure 1), which contain phage replicase, recombinase, integrase, and mobile genetic elements. Aside the differences in gene order, the virulence genes profile in the C6 isolate is similar with the *V. cholerae* O395 but not the N16961 and the other vibrio isolates from this study (Supplementary Table 3).

In isolates E30 and E32, the genomic region containing the *tcp* and *acf* gene clusters was replaced by genes encoding the type I restriction-modification system subunits (restriction subunit, DNA-methyltransferase, and specificity subunits), a type III restriction enzyme, a putative endonuclease, and DNA-integration/recombination/inversion protein (Figure 2A). Similarly, the *tcp* and *acf* region in E4 was replaced with genes encoding the type I-F CRISPR-Cas system (Figure 3A). The replacement of the virulence genes with phage defense mechanisms such as the restriction-modification and the CRISPR system, reflects the requirement for survival in the external environment whereby bacteria are more prone to phage attack. However, interfering with plasmids acquisition and phage infections comes at the cost of inhibiting the acquisition of beneficial genes such as antibiotic resistance and virulence factors (Watson et al., 2018).

In the environmental isolate S1, the genomic region containing the *zot* and *ctx* toxin genes was replaced by the bacteriophage F237 protein genes, the bacteriophage DNA epimerase, and the *rstAB* genes (Figure 2C). This region in S1 also possesses a *zot* gene encoding a protein that shares almost 100% amino acid sequence identity with the Zot protein in *Vibrio albensis* VL426 but only 60% amino acid identity with the N16961.

Despite absence of the *ctx* and *zot* in E4, E19, and S35, the order of genes flanking the IS3-ISVch4 element and the CTXΦ *rstC* gene was similar to that of the N16961 reference strain (Figure 2C). Comparison of the S35 sequences with the *tcp* and *acf* clusters of N16961 revealed that it is inserted in the region containing an integrase and IS481 insertion element, however, in S35, the gene order in the regions flanking the integrase and insertion elements was conserved (between 88 and 100% homology) (Figure 2C). Also, comparison with the sequence from the non-O1 isolate *V. cholerae* O77 RIMD reveals the possibility of integration or excision of a phage elements in the region (Figure 2C).

Recent trends show the emergence of antimicrobial resistance in *V. cholerae* mostly due to the acquisition of MGEs from closely or distantly related strains (Das et al., 2020). Analysis of antimicrobial resistance genes did not show a clear distinction between the clinical and environmental strains (Supplementary Table 4). Further, the distribution of antimicrobial resistance genes was not limited by the spatial and temporal distribution of the isolates, as some environmental strains possessed some antimicrobial resistance genes which were present in some clinical isolates and vice versa (Supplementary Table 4).

Against the backdrop of virulence and antimicrobial resistance genes acquisition, the presence of MGEs such as Integrative and Conjugative elements (ICEs) serve as conduits for the dissemination of adaptive traits among bacterial populations. ICEs are composed of core gene sets encoding T4SS-based conjugation apparatus, integrase or resolvase, and accessory genes. Our analysis shows that seven isolates contained ICEs ranging from 100 to 270 kb with defined direct repeats (Supplementary Figure 5). Aside from the C1, which shared homology with a portion of genes in the ICEs identified in the environmental isolates E30 and E32, most gene contents conserved in the ICEs of the clinical isolates were absent in those of the environmental isolates (Supplementary Figure 5). However, within each group, there was high homology and synteny of the genes harbored in the ICEs (Supplementary Figure 5). These genetic similarities in the ICEs identified within both groups of isolates confirm previous studies indicating that when present in the environment, ICEs can spread in bacterial communities and provide an adaptive advantage for that environmental niche.

### Genome plasticity in the *Vibrio cholerae* isolates

Genome plasticity is paramount for the evolutionary adaptation of pathogens to their environment (Dobrindt and Hacker, 2001; Chaguza et al., 2015). The accumulation of pathoadaptive mutations driving the evolution of pathogenic variants from non-pathogenic ancestors is vital for the success of colonization and infection (Keymer and Boehm, 2011). To investigate the level of genome plasticity and recombination, we performed genome-wide alignment of the isolates around their origin of replication using the N16961 as a reference. The results revealed higher scores for chromosomal inversions, breakpoints, and translocation within the environmental isolates than in the clinical isolates (Table 2 and Supplementary Figure 6).

**FIGURE 4:**
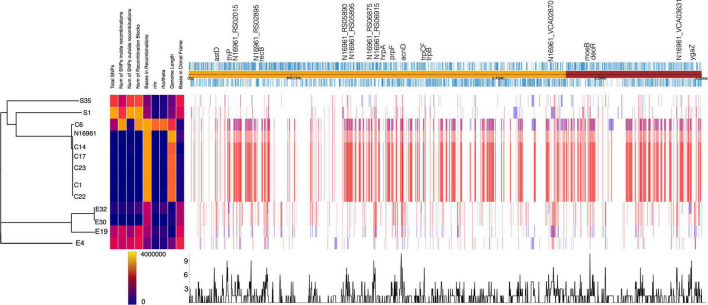
Homologous recombination events detected in *V. cholerae* strains. The phylogenetic tree is generated from the number of SNPs. The homologous recombination events are shown by blocks, which are colored blue for unique events in strains and red for events occurring in multiple strains. The recombination hotspots are marked at the top of the plot.

To further understand the mechanisms underpinning the differences in genomic rearrangement patterns among the isolates, we investigated the presence of genes that influence recombination. The analysis revealed that the *recD*-like DNA helicase *yrrC* was present only in the S35 isolate. The DNA repair and error-prone DNA repair system *umuCD* was absent in the environmental isolates E30, E19, E32, E4, and S35, and the C6 clinical isolate. The PolV (UmuD2′C) encoded by the *umuCD* operon enhances bacterial survival upon DNA damage by ensuring DNA replication through lesions that block replication forks (Sutton et al., 2000). It possible that an absent or inactive UmuCD system accounts for the numerous breakpoints (indels, MNPs, and SNPs) identified in the genome of the environmental isolates. Next, we examined in the isolates the genomic regions associated with high levels of recombination events, which highlight genes under diversifying selective pressure (Figure 4). The overlap in the recombination frequency of each gene was calculated using Gubbins. We identified about 40 hotspots with high recombination frequency in more than half of the genomes (Figure 4 and Supplementary Table 5). Most of these hotspots occurred in genes involved in nucleotide metabolism including DNA repair (*recB*, *hrpA*, *ada*), and DNA (*deoR*) and RNA metabolism (*astD*). This agrees with the notion that high recombination events in the *recBCD* cluster are involved in recovering broken replication forks *via* homologous recombinational DNA repair (Dillingham and Kowalczykowski, 2008).

Two genes with the highest recombination frequencies were *acnD* and *prpF*, which are involved in the methylcitrate cycle for propionate metabolism and fatty acid catabolism, respectively (Supplementary Table 5 and Supplementary Figure 8). This pathway allows bacteria to utilize bactericidal short-chain fatty acids such as propionate as carbon and energy sources. The evolution of bacteria to degrade propionate, which is abundant in the soil and human intestines, highlights the adaptive mechanism of *V. cholerae* to survive in the environment and cause diarrhea upon colonization of the human gut (Chaguza et al., 2015).

### Phage and plasmid defense systems the *Vibrio cholerae* isolates

The CRISPR-Cas system is an adaptive immune mechanism used by bacteria to maintain genome integrity by interfering with the insertion of deleterious foreign sequences from phages or *via* HGT. We identified the CRISPR-Cas system in one clinical (C6) and three environmental (S1, E4, and E19) isolates (Table 1). The CRISPR-Cas systems occurred in sporadic locations in the genomic regions containing mobile genetic elements and phage proteins, implying acquisition *via* HGT and not vertically transfer. Also, all *cas* genes identified were intact (Supplementary Table 2) and therefore likely functional.

**FIGURE 5:**
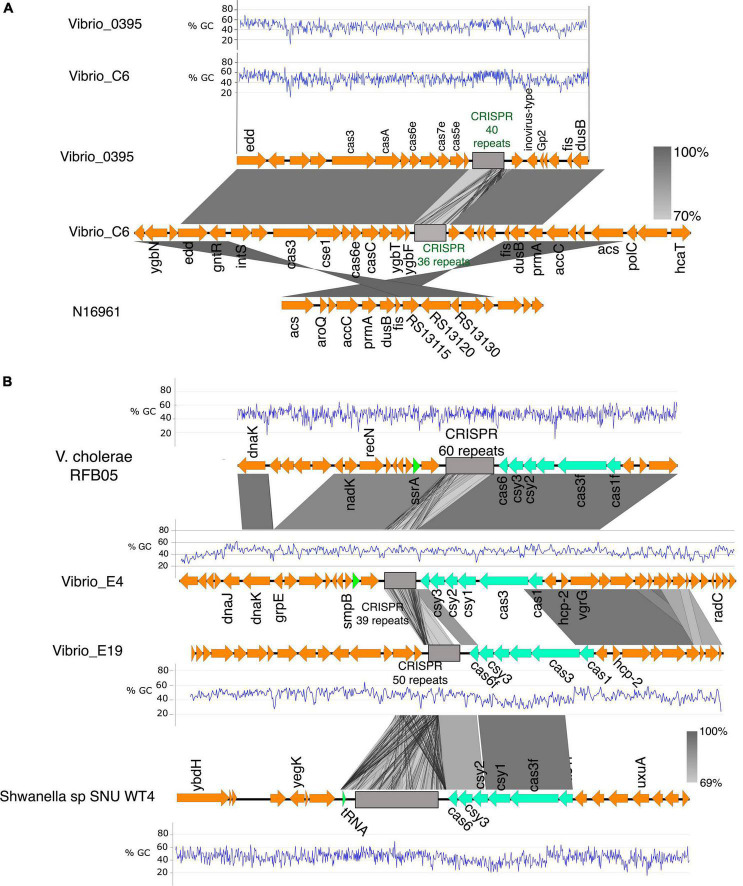
The CRISPR-Cas system in *V. cholerae* isolates. **(A)** The CRISPR-Cas system and alignment of the region in the clinical isolate C6, *V. cholerae* 0395, and the N16961. The C6 harbors a type I-E CRISPR-cas system homologous to the one in *V. cholerae* 0395 with similar GC% patterns of the *cas* genes. The region flanking the CRISPR-Cas system in the C6 is conserved in the N16961. **(B)** Genetic organization of the CRISPR-Cas loci in the environmental isolate E4 and E19 and their alignment with *V. cholerae* RFB05 and *Shewanella sp SNU WT4*. The type I-F CRISPR-Cas system in E4 is homologous to the one in *V. cholerae* RFB05. The type I-F CRISPR-Cas system in the E19 is homologous to the one in *Shewanella sp*. SNU WT4 and shares almost similar GC%, which is anomalously low and probably acquired by lateral gene transfer (%GC of Cas genes in E19 is 41% compared to the overall GC% of 47.7% in E19. The grayscale bars show the nucleotide homology in the aligned regions.

Sequence analysis and comparison of the CRISPR-Cas locus located in Chromosome 1 of *Vibrio*_C6 revealed homology with the *V. cholerae*_0395; however, the latter contained 40 CRISPR repeat units whereas C6 has 36 repeat units in its CRISPR array. The genes flanking the CRISPR-Cas system in both strains were closely matched (Figure 5A) with similar patterns in GC contents. Among the genes directly flanking the CRISPR-Cas systems in the two bacteria are the *insT* (site-specific integrase) and the phage Gp2 proteins. The C6 contained the *cas3, cse1, casB, cas6e, casC, cas5, cas1*, and *ygbF* gene cluster followed by the CRISPR array, which are components of the Type I-E CRISPR-Cas system (Figure 5A). The C6 CRISPR-Cas system is in the genomic island and is absent in N16961.

The environmental isolates E4 and E19 contain the type I-F CRISPR-Cas system comprising the gene cluster *cas1, cas3, csy1, csy2, csy3*, and *cas6f* with 39 and 50 repeat units of the CRISPR array, respectively. The CRISPR array of the E4 is flanked by the tyrosine-type recombinase and DNA repair protein RecN which share high sequence homology (95.94% sequence homology of the *cas* genes) with the type I-F CRISPR-Cas system and the neighboring genes in *V. cholerae* RFB05 (Figure 5B). There was a clear difference in the GC content in the CRISPR-Cas components and neighboring genes of the E4 and E19 (E4:E19, 21% sequence identity of the Cas genes; GC% E4 = 46, and E19 = 41) (Figure 5B). The neighboring genes in the CRISPR-Cas system region of E19, share conserved synteny with that of *V. cholerae* O51 RIMD 2214289; however, sequence homology between the CRISPR-Cas systems in the two genomes was very low (19% homology in Cas genes). By performing a global search using the CRISPR arrays and the Cas genes in E19, we identified high sequence homology (95% homology of the Cas genes; GC%; E19 = 41% and SNU WT4 = 41%) with the type I-F CRISPR-Cas of the *Shewanella* sp SNU WT4, which was isolated from an infected rainbow trout (Figure 5B).

E19 also possesses another CRISPR array with 10 repeats but no associated *cas* genes located 47 kbp upstream of the type I-F CRISPR system (Supplementary Figure 7). The region between these two genes comprises genes encoding toxin-antitoxin systems, a multidrug resistance transporter, ATP synthases, streptomycin resistance, and hypothetical proteins. The two CRISPR arrays are located in the GI-2 (integron) (Supplementary Figure 1B) region, which has a high degree of gene capture and excision and occupies about 3% of the *V. cholerae* genomes (Orata et al., 2015).

**FIGURE 6:**
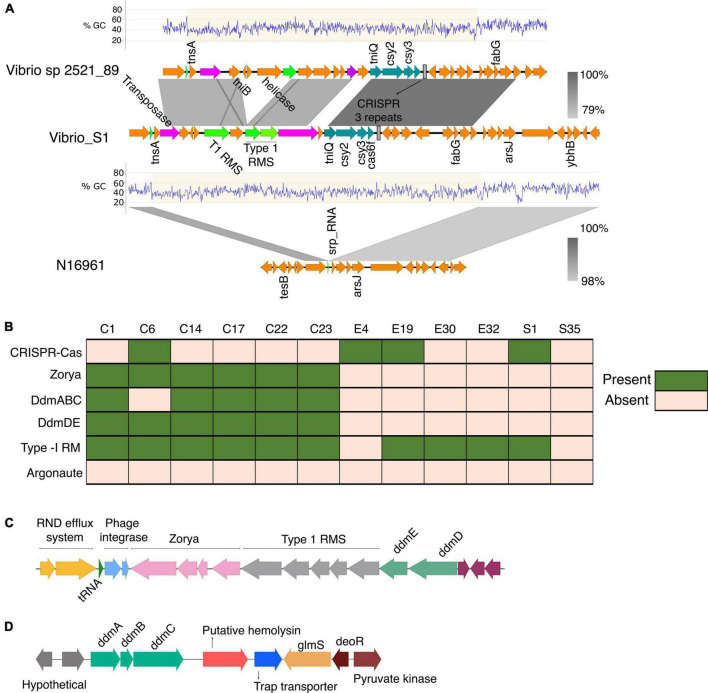
**(A)** Genetic organization of the CRISPR-Cas system identified in the *V. cholerae* isolate S1. The Tn*7*- like transposon regions with the associated cargo carrying the mini type I-F CRISPR-Cas system in both *V. cholerae* isolate S1 and the *Vibrio* sp 2521_89 were aligned and compared using BLAST and EasyFig. The Tn*7* element is inserted into the site downstream of the signal recognition particle RNA (srp_RNA). **(B)** The distribution of phage defense mechanisms in the clinical and environmental vibrio isolates. **(C)** Schematic of the gene cluster containing the phage and plasmid defense components commonly found in VP_II. The gene order is representative of the clinical isolates from this study and the N16961. **(D)** Schematic of the *ddmABC* gene cluster in the *V. cholerae* clinical isolates.

The isolate S1 contained the mini type 1-F CRISPR-Cas system comprising the gene cluster *tniQcsy2csy3cas6*, which shared high sequence homology with the CRISPR-Cas system found in the *V. cholerae*_2521-28 (Figure 6A). *tniQ* forms an operon with *csy2, csy3*, and *cas6* and it product is similar to TnsD, which targets an *attTn7* site for insertion (Sharpe and Craig, 1998; McDonald et al., 2019). The CRISPR array in both systems has three repeats typical for the mini CRISPR-Cas system found in other vibrio strains (McDonald et al., 2019). The DNA region around the min type I-F CRISPR-Cas shows a 36-kb anomalously lower GC content, which is indicative of horizontal gene transfer (%GC content: 42% compared to 47% across the entire genome). In the S1, the type 1 restriction-modification system is associated with the Tn*7*-like element whereas the *Vibrio* sp. 2521_89 DNA region contains cargo genes encoding the type 1 restriction-modification systems (RMS), multidrug efflux system, and helicase (Figure 6A). In the type 1 RMS, the Tn*7* element is inserted into the site downstream of the signal recognition particle RNA (srp_RNA) (Figure 6A). The presence of such cargo genes is common in *V. cholerae* strains that possess this type of Tn*7*-transposons (McDonald et al., 2019).

CRISPR arrays contain spacer sequences acquired from previously encountered phages or mobile genetic elements that provide memory to protect against future infections. To understand the adaptation and specificity of the CRISPR-Cas systems present in the isolates we examined the spacer homology in the phage and plasmids databases using CRISPRTarget. Common target protospacers in *Vibrio* phages were identified in the type I-E and I-F CRISPR-Cas systems present in C6, E4 and E19. The *Vibrio* C6 and E19 spacers have target protospacers in the CTXΦ and other *Vibrio* phages (Supplementary Table 6). The S1 mini Type I-F CRISPR-Cas system with two spacers was homologous to targets present only in plasmids (Supplementary Table 6), which is typical of *Vibrio* isolates possessing the mini type I-F CRISPR-Cas system that targets protospacers mostly in plasmids, prophages and a few lytic phages (McDonald et al., 2019). The presence of intact type I-E and I-F Crispr-Cas components in the isolates can diminish their capacity to acquire the *ctx* toxin gene from the ssDNA CTXΦ phage since the type I-E and I-F CRISPR-Cas system target and cleave foreign ssDNA (Makarova and Koonin, 2015). Acquisition and maintenance of a functional CRISPR-Cas system in the isolates confer an essential adaptive trait for survival in the external environment where bacteria are outnumbered by phages and exposed to environmental DNA.

Despite the HGT acquisition of beneficial traits *via* phages, and mobile genetic elements, exogenous genetic materials can carry integrative elements which disrupt genome integrity. Additionally, the acquisition of non-beneficial genes can affect the fitness of strains and reduce their competitive advantage in polymicrobial settings. Notably, all the isolates from this study, as most of the pandemic strains (Jaskólska et al., 2022), lack plasmids. Hence, we investigated possible factors underpinning the absence or stability of plasmids in the isolates and its evolutionary implications. A BLAST search showed the presence of the defense systems Zorya, Type-1 Restriction Modification (RM) system, and the VC1770-VC1771(DdmDE) (Doron et al., 2018; Jaskólska et al., 2022) arranged in tandem in the VPI-2 of the clinical isolates (Figures 6B,C). Also, the VC0490-VC0492 (DdmABC) was found in the VSP-II of the clinical isolates except for the classical strain C6 (Figure 6D). As reported for other classical *Vibrio* strains, C6 lacks the *ddmABC* that enhances *ddmDE* plasmid degradation activity (Jaskólska et al., 2022). The absence of plasmids in the environmental isolates implies the potential functionality of the Type I RM systems identified in four isolates. However, the absence of similar defense systems in the S35 isolates suggests an alternative mechanism for phage defense or plasmid acquisition and stability in the bacteria. Aside from S1 with the type 1 RM system on a Tn*7*-like element (Figure 6A), the other environmental isolates contained two type-1 RM systems located in different regions in the genome.

## Discussion

The detailed comparative genome analysis of *V. cholerae* isolates from Ghana provided in this study, which includes genomic complexities influencing the adaptive evolution of bacteria such as SNPs, recombination, pseudogenization, acquisition of virulence and antimicrobial resistance determinants, and defense against deleterious foreign genetic elements, reveals that various aspects of the bacterial genome differ between the clinical (outbreak) and environmental isolates.

Concerning the acquisition of genetic materials, the distribution of phage and plasmid defense systems in the clinical and environmental isolates (Figures 5, 6) highlights the differences and ability of environmental strains to acquire genetic elements from the environment to drive their adaptation. Particularly, the distribution of phage and plasmid defense mechanisms in the isolates may explain why plasmids are abundant in environmental strains but rare in pandemic strains (Jaskólska et al., 2022), as well as the possibility of environmental strains acquiring and maintaining plasmids with beneficial traits and virulence determinants.

The presence SNPs and orthologs among the Vibrio isolates revealed substantial genetic diversity and clustering among the isolates in terms of their source of isolation and biotypes (Figure 1). Additionally, from a combination of SNP frequencies, and recombination patterns (Figure 4), we observe how the clinical and environmental isolates diverge in distinct groups with the environmental strains possessing higher frequency of SNPs in recombination blocks.

The comparative analysis of the genome architecture of *V. cholerae* environmental and clinical isolates in this study indicate that *V. cholerae* strains undergo extensive genomic rearrangements coupled with gene acquisition, illustrating their adaptation during ecological shifts and pathogenicity. We provide results that refine and expand our knowledge about the differences in the evolution of virulence determinants, genomic rearrangement, and defense mechanisms by environmental and clinical *Vibrio* isolates. *V. cholerae* genomes undergo significant changes depending on the bacterial lifestyles in native aquatic environments or infection. Although it is believed that clinical isolates are progenitors of environmental strains, it remains unclear how environmental isolates acquire virulence genes encoding *tcp*, *ctx*, *zot*, and *acf via* HGT to initiate another epidemic. The observed differences in evolutionary divergence through decay in the ortholog conservation and genetic alterations among the environmental isolates compared to the clinical isolates shows a more progressive evolution of bacteria in the environment. The transfer of virulence determinants by HGT *via* phages or mobile vectors could enable a transition to a pathogenic lifestyle, but it is also modulated by the evolving nature of adaptive mechanisms to selectively interfere with the acquisition of exogenous genetic material. The homology of the CRISPR-Cas gene clusters in the isolates with those from different bacterial strains indicates recent HGT and implies that the transfer of adaptive traits among bacteria is an ongoing process. The acquisition of the CRISPR-Cas system and restriction modification system reflects the acquisition of traits that may be critical for bacterial fitness either when competing in a polymicrobial setting (e.g., the gut microbiota) or in its native aquatic environment. Conversely, the CRISPR-Cas-mediated interference with the entry of foreign genetic material can reduce the acquisition of beneficial genes since the CRISPR spacers in the isolates share homology with protospacer targets in common *Vibrio* phages and plasmids. It is anticipated that the genomic differences between the clinical and environmental isolates determine the bacterial survival outcomes (infection or niche adaptation in the native aquatic environment). However, it remains less clear how the bacteria transition from a non-pathogenic to a pathogenic lifestyle.

## Data availability statement

The data presented in this study are deposited in the NCBI repository, accession numbers SAMN26804330, C23; SAMN26804331, C22; SAMN26804328, C14; SAMN26804326, C1; SAMN26804327, C6; SAMN26804329, C17; SAMN26804320, S1; SAMN26804321, S35; SAMN26804322, E4; SAMN26804323, E19; SAMN26804324, E30, and SAMN26804325, E32.

## Author contributions

YA, SD, and SA designed the experiment. NA, LM, and SD conducted the experiment. NA, MV, and SA performed data analysis. MV, NA, and SA wrote the manuscript. All authors contributed to the article and approved the submitted version.
