# Supplementary material for: Comparative analysis of Vibrio cholerae isolates from Ghana reveals variations in genome architecture and adaptation of outbreak and environmental strains
Source: Front Microbiol. 2022 Oct 13;13:998182. doi: 10.3389/fmicb.2022.998182 (PMC9608740; doi:10.3389/fmicb.2022.998182)
Supplement: Supplementary file 1 [file Data_Sheet_1.PDF]

Fig S1

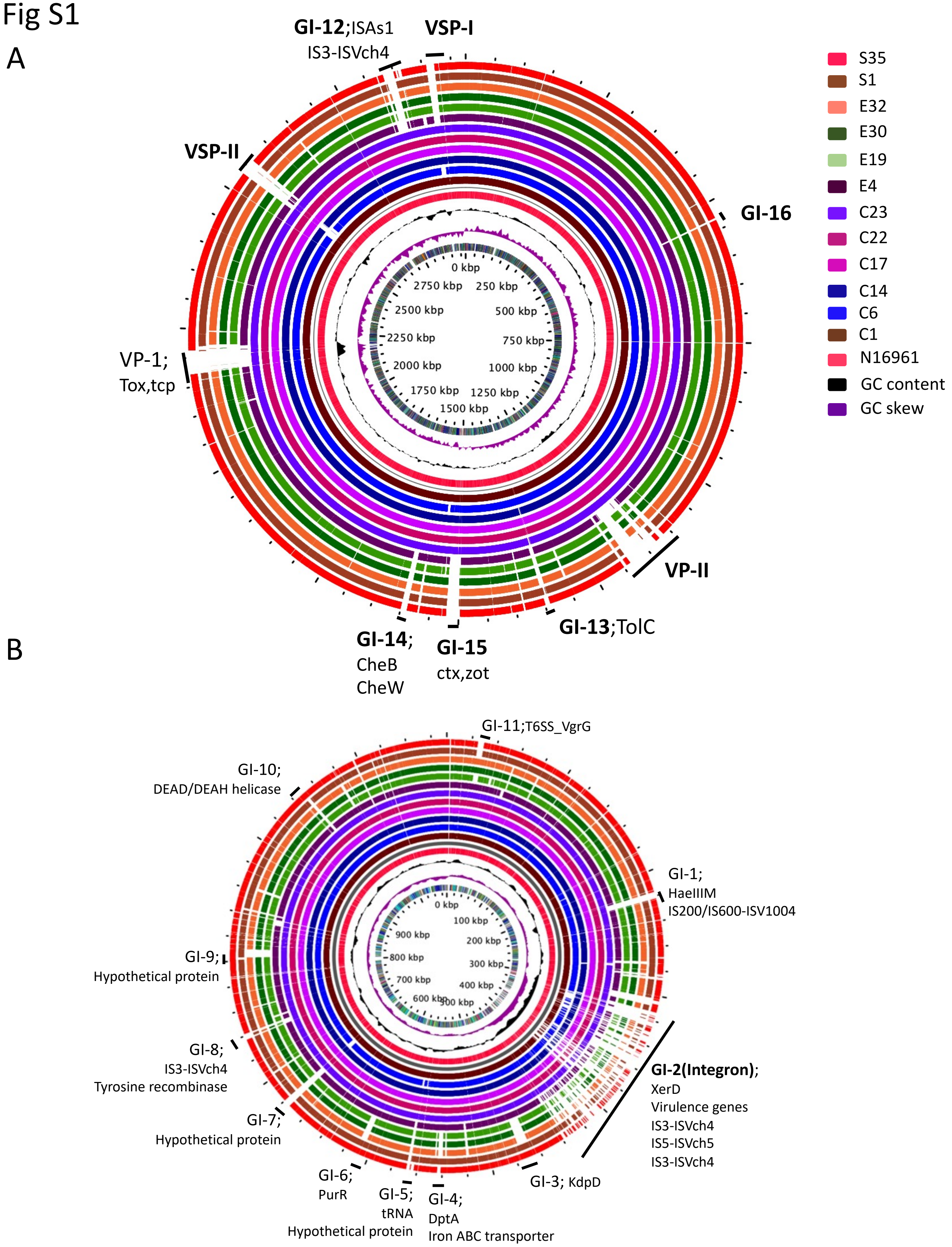

Fig. S1. Schematic representation of the *Vibrio cholerae* genomes alignment for **A**) chromosome I and **B**) chromosome II. Allelic ORFs in the aligned genomes are colored according to the predicted COG categories (inner circle). Genomic island (GI) predicted with Islandviewer are shown for the two chromosomes with some genes commonly found in the genomic regions are listed with the GIs.

Figure S2

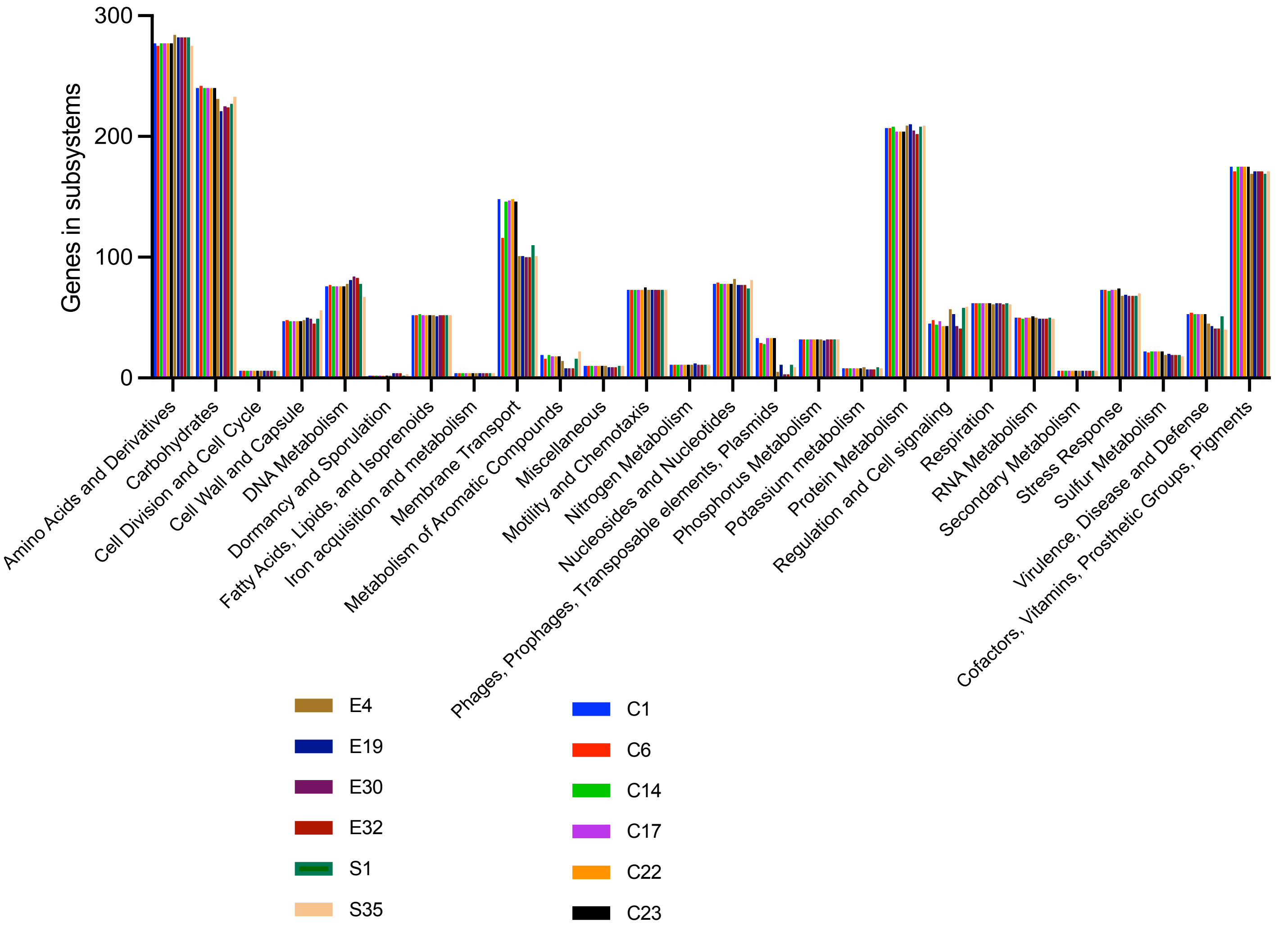

Fig. S2. Subsystem categorisation of genes present in the *V. cholerae* isolates.

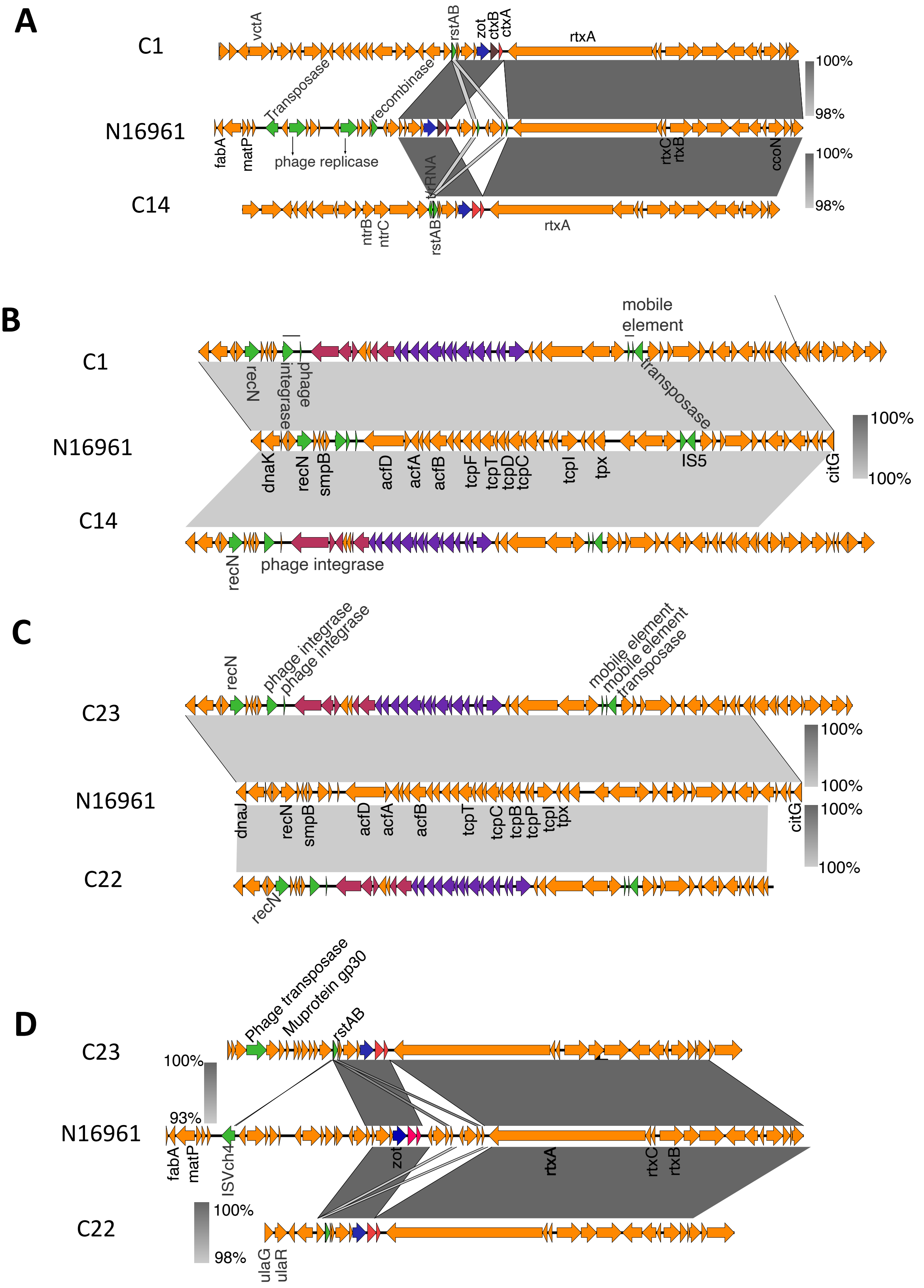

Fig. S3. Virulence gene in *V. cholerae* clinical isolates. The genetic organization and alignment of the **A**. ctx and zot and **B**. acf and tcp gene region in the clinical isolates C1, C14 and N16961. **C**. The alignment of the acf, and tcp region and **D**. the zot and ctx of the clinical isolates C22, C23 and N16961. The grayscale bars show the nucleotide homology in the aligned regions.

Figure S4

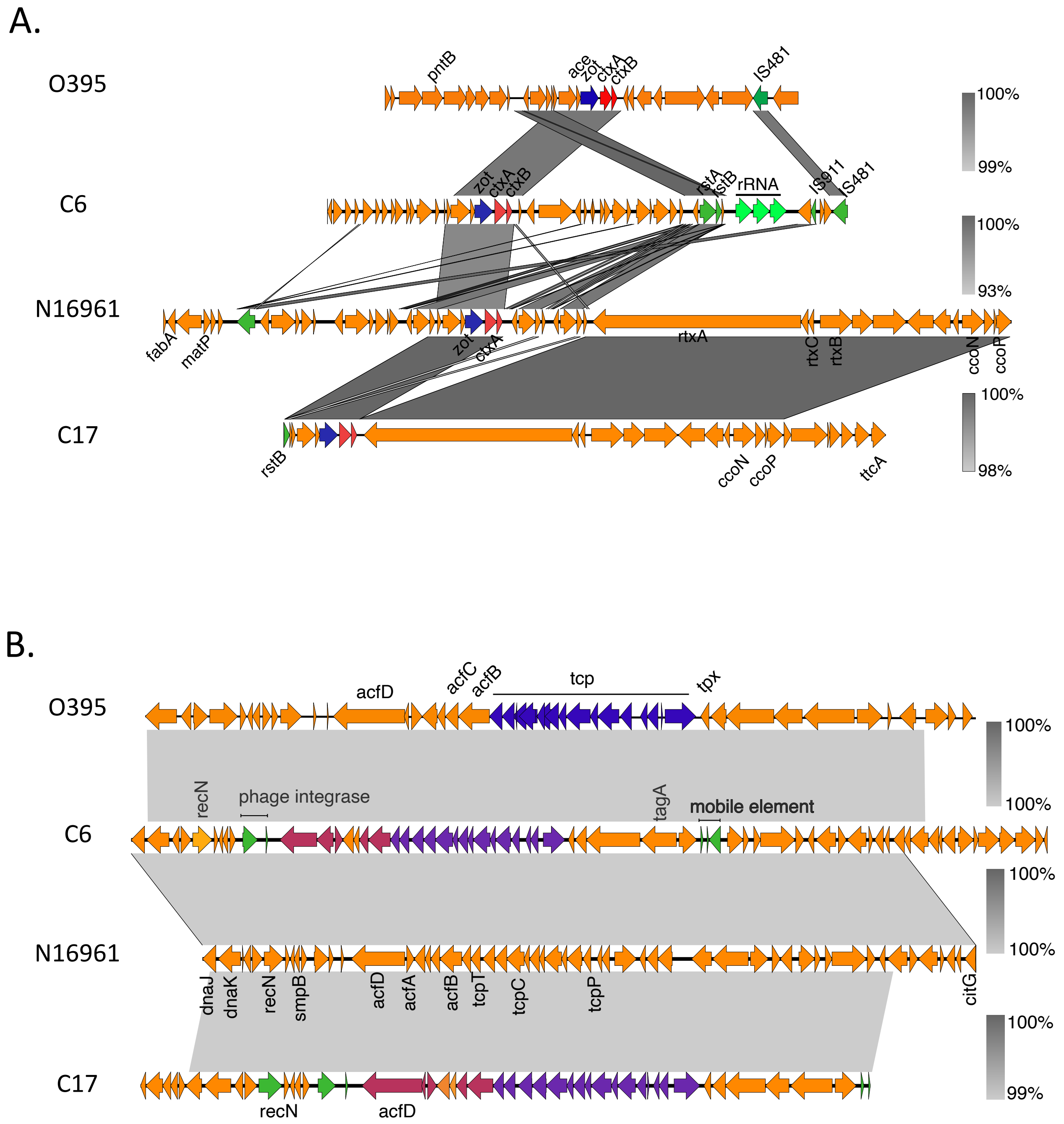

Fig. S4. Virulence gene in *V. cholerae* clinical isolates. **A**. The comparative alignment of the zot and ctxAB region C6, C17, N16961 and O395. **B**. The comparative alignment of the acf and tcp region in C6, C17, N16961 and O395. The grayscale bars show the nucleotide homology in the aligned regions.

Figure S5

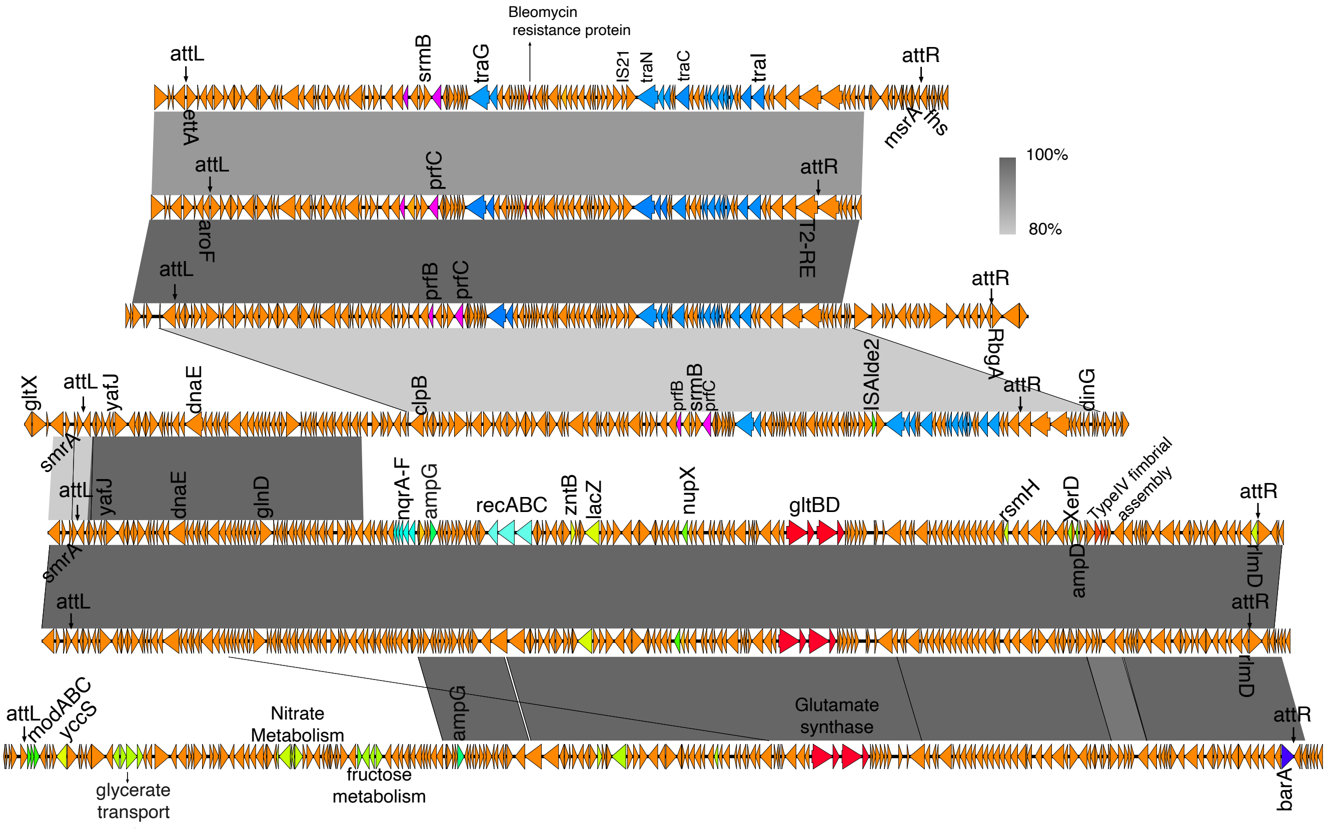

Fig S5: Comparison of Integrative and conjuagative elements ICEs in the Vibrio cholerae clinical and environmental isolsates.The direct repeats and the positions delimiting the ICEs are represented by attL and attR. The grayscale bars show the nucleotide homology in the aligned regions.

Figure S6

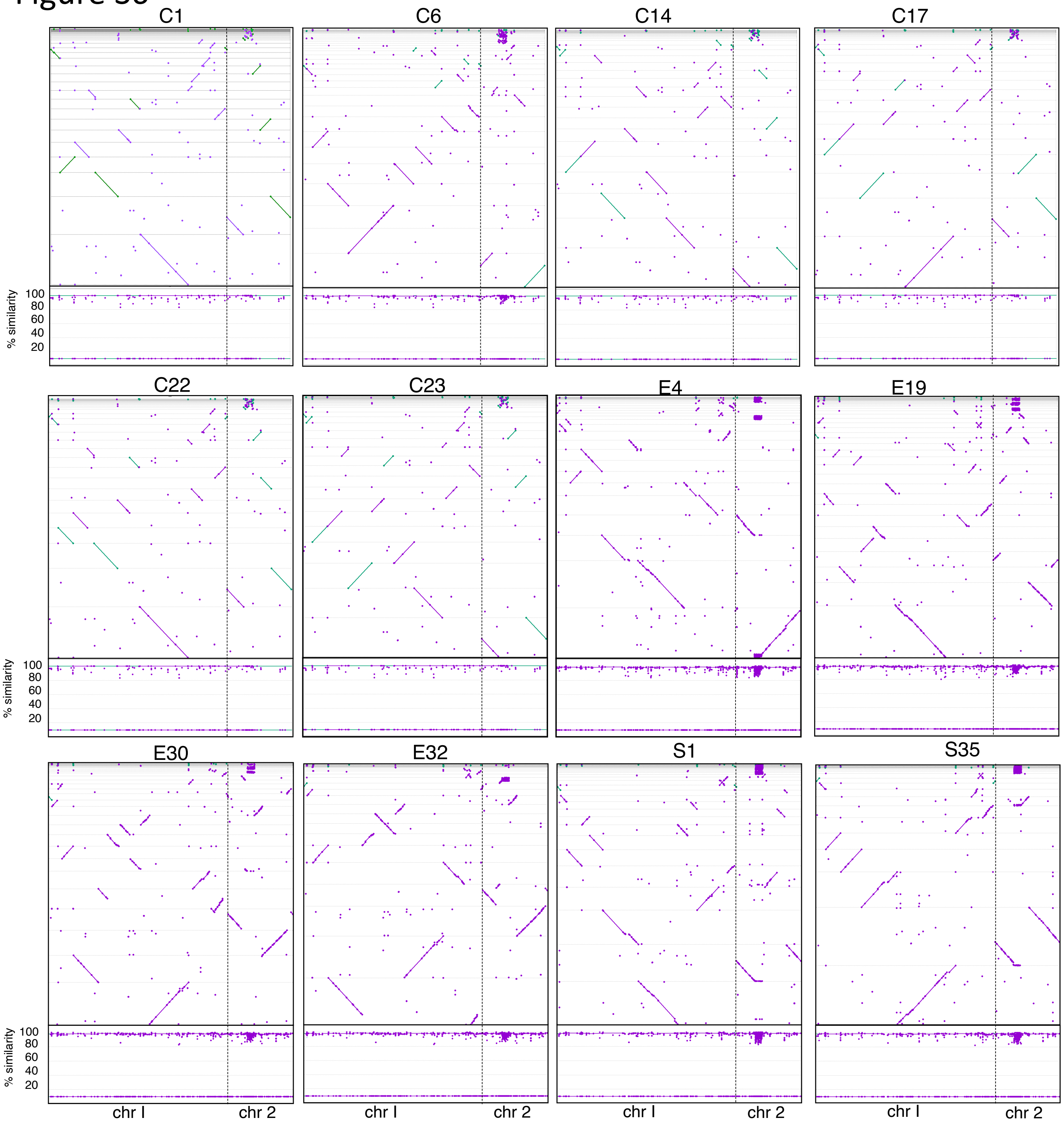

Fig S6. Whole genome nucleotide alignment and percentage similarty of the *V. cholerae* isolates with the reference strain N16961. The plots represent the maximal unique matching forward and reverse sequences between the genomes of the *V. cholerae* isolates and the N16961. A point (x,y) indicates 100bp nucleotide cluster that occur within each genome once at location x in one and at location y in the other genome. The coverage plot shows the percentage similarity between the nucleotide sequences of the isolates and the N16961. A line of dots with slope of 1 represents an undisturbed segment of conservation between the two sequences, while a line of slope of -1 represents an inverted segment of conservation between the two sequences. Negative slope segments in the upper left quadrant of the graph shows both an inversion and translocation. Segments located in the upper right quadrant with negative slope of the graph show only inversions.

Figure S7

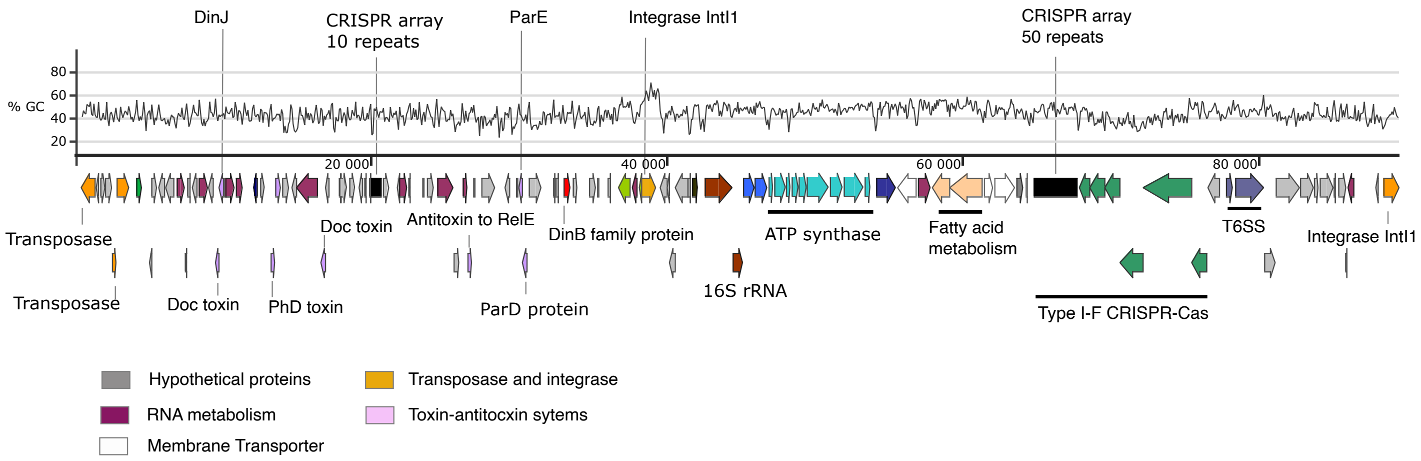

Fig. S7.CRISPR-Cas systems in *V. cholerae* isolates. Genomic region of E19 containing the type I-F CRISPR-Cas system and a 10-repeat sequence CRISPR array found approximately 47 kb apart.

Figure S8

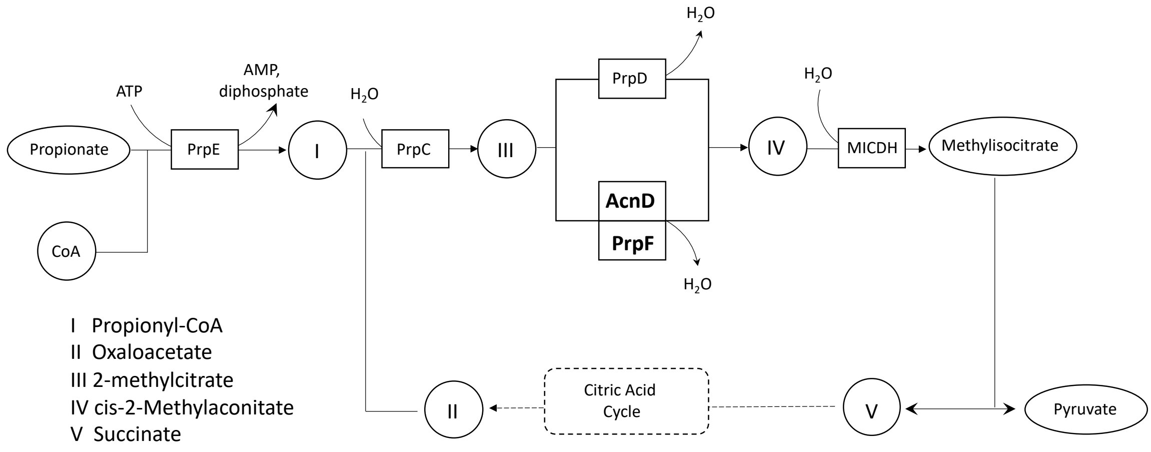

Fig. S8. Schematic representation of the pathway involving PrpF and AcnD for the breakdown of propionate to 2-methylcitrate.
